# Association of sodium-glucose cotransporter 2 inhibitors with the incidence of corneal diseases in type 2 diabetes mellitus

**DOI:** 10.7150/ijms.91571

**Published:** 2024-01-20

**Authors:** Tsan-Yu Tsai, Po-Jen Yang, Shih-Chun Chao, Chia-Yi Lee, Jing-Yang Huang, Shun-Fa Yang, Hung-Yu Lin

**Affiliations:** 1 Department of Ophthalmology, Show Chwan Memorial Hospital, Changhua, Taiwan.; 2 Department of Family and Community Medicine, Chung Shan Medical University Hospital, Taichung, Taiwan.; 3 School of Medicine, Chung Shan Medical University, Taichung, Taiwan.; 4 Department of Optometry, Central Taiwan University of Science and Technology, Taichung, Taiwan.; 5 Department of Optometry, Yuan Pei University, Hsinchu, Taiwan.; 6 Institute of Medicine, Chung Shan Medical University, Taichung, Taiwan.; 7 Department of Ophthalmology, Nobel Eye Institute, Taipei, Taiwan.; 8 Department of Ophthalmology, Jen-Ai Hospital Dali Branch, Taichung, Taiwan.; 9 Department of Medical Research, Chung Shan Medical University Hospital, Taichung, Taiwan.; 10 Department of Post-Baccalaureate Medicine, College of Medicine, National Chung Hsing University, Taichung, Taiwan.; 11 Department of Optometry, Chung Shan Medical University, Taichung, Taiwan.

**Keywords:** SGLT2 inhibitors, epidemiology, superficial keratopathy, infectious keratitis, age

## Abstract

Sodium-glucose cotransporter 2 (SGLT2) inhibitors revealed the protective function on various systemic diseases. This study aimed to determine whether the usage of SGLT2 inhibitors associates with incidences of superficial keratopathy and infectious keratitis in type 2 diabetes mellitus (T2DM) patients. A retrospective cohort study with the usage of National Health Insurance Research Database of Taiwan was conducted. The T2DM patients were divided into the SGLT2 inhibitors and control groups according to the usage of SGLT2 inhibitors or not. The major outcomes were defined as the occurrence of superficial keratopathy and infectious keratitis. There were 766 and 1037 episodes of superficial keratopathy in the SGLT2 inhibitors and control groups and SGLT2 inhibitors group showed a significantly lower incidence of superficial keratopathy than the control group (aHR: 0.721, 95% CI: 0.656-0.791, P < 0.0001). Also, there were 166 and 251 infectious keratitis events in the SGLT2 inhibitors and control groups and patients in the SGLT2 inhibitors group revealed a significantly lower infectious keratitis incidence than those in the control group (aHR: 0.654, 95% CI: 0.537-0.796, P < 0.0001). In addition, the patients that received SGLT2 inhibitors demonstrated lower cumulative incidences of both superficial keratopathy and infectious keratitis compared to the non-SGLT2 inhibitors users (both P < 0.0001). In conclusion, the usage of SGLT2 inhibitors correlates to lower incidence of superficial keratopathy and infectious keratitis in T2DM individuals, which is more significant in patients with persistent SGLT2 inhibitors application.

## Introduction

The type 2 diabetes mellitus (T2DM) is a metabolic disorder that manifested with hyperglycemia and with a worldwide prevalence above 8 percent 1. About the treatment of T2DM, the oral anti-hyperglycemic medications and insulin injection are common methods 2. The sodium-glucose cotransporter 2 (SGLT2) inhibitors have been used to control the hyperglycemia in T2DM individuals with fair outcome 3, 4. In the previous publication, the usage of SGLT2 inhibitors on T2DM patients who already took the dipeptidyl peptidase-4 inhibitor monotherapy can reduce the glycated hemoglobin level of about 0.71% 5.

In addition to the anti-diabetic effectiveness, the SGLT2 inhibitors also revealed protective function on certain other organs 3, 6. The SGLT2 inhibitors in the diabetic mice would reduce the cardiomyocytes autosis and the associated myocardial infarction episode 7. In addition, the SGLT2 inhibitors utilization could reduce the heart failure incidence in previous study written by Karagiannis *et al* 8. Except for the cardiovascular morbidities, the SGLT2 inhibitors can decrease the incidence of hyperkalaemia and anaemia in individuals diagnosed with the chronic kidney disease 9. Consequently, the SGLT2 inhibitors might demonstrate protective function in other disorders with similar pathophysiology.

The association between SGLT2 inhibitors and ophthalmic disorders had been discussed in previous literatures 10, 11. Both the experimental and clinical studies revealed that the usage of SGLT2 inhibitors have partial benefit on the development and progression of diabetic retinopathy 12-14. Another two researches showed the protective effect of SGLT2 inhibitors on the improvement of dry eye disease 15, 16. However, there was scant research to evaluate the relationship between SGLT2 inhibitors and corneal diseases. Because both corneal diseases and dry eye disease occur on the ocular surface and can be treated by same agent 17, the SGLT2 inhibitors might also own protective effect on corneal diseases.

The purpose of our study is to investigate the potential correlation between the SGLT2 inhibitors application and the development of corneal disease including superficial keratopathy and infectious keratitis via the data in National Health Insurance Research Database (NHIRD). Other risk factors for the ocular surface diseases and severity indexes for T2DM were also included in our analysis model.

## Materials and Methods

### Data source

This research was approved by both the National Health Insurance Administration of Taiwan and Institutional Review Board of Chung Shan Medical University Hospital (Project code: CS1-20108). NHIRD includes insurance-claimed data of practically 23 million residents of Taiwan with time period from January 1, 2014 to December 31, 2020. The accessible data in the NHIRD involves International Classification of Diseases-Ninth Revision (ICD-9), International Classification of Diseases-Tenth Revision (ICD-10) diagnostic codes, sex, age, education level, urbanization level, income level, type of insurance, image exam codes, laboratory exam codes, medical department codes, procedure codes, surgery codes and the international ATC codes for pharmaceutical.

### Participant Selection

**Figure 1:**
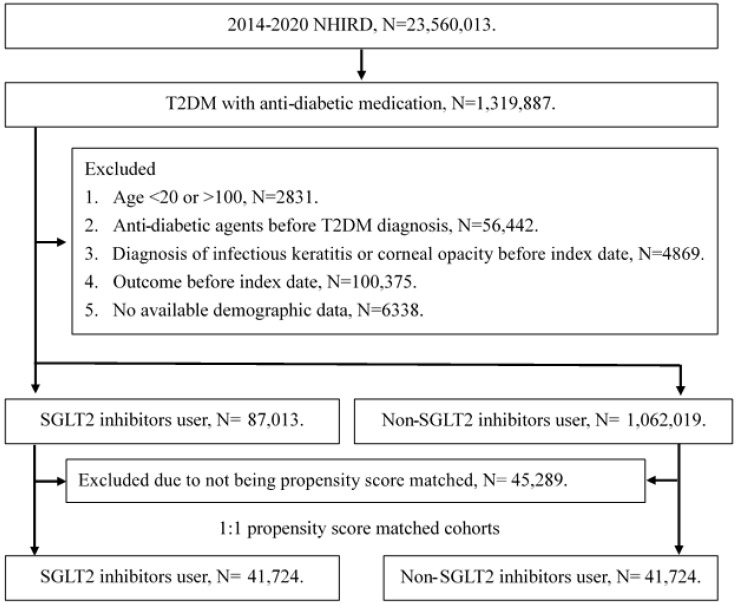
** The flowchart of participant selection.** NHIRD: National Health Insurance Research Database, N: number, T2DM: type 2 diabetes mellitus, SGLT2: sodium-glucose cotransporter 2.

A retrospective cohort study was conducted and individuals with T2DM were defined as SGLT2 inhibitor users that satisfied the following criteria: (1) receipt of T2DM diagnosis based on ICD-9 or ICD-10 codes from 2014 to 2019, (2) had visited internal medicine or family medicine administration with follow up interval longer than three months, and (3) the application of SGLT2 inhibitors prescriptions including empagliflozin, dapagliflozin, canagliflozin and ertugliflozin via the international ATC codes. The index date of the current study was set as date 6 months after the start of SGLT2 inhibitors therapy. Also, the following exclusion criteria were used to standardize the T2DM population: (1) loss of demographic data, (2) application of anti-diabetic medication before T2DM diagnosis, (3) individuals aged younger than 20 years or older than 100 years, (4) the presence of corneal opacity before the index date, and (4) the presence of superficial keratopathy or infectious keratitis before the index date. After that, each participant in the SGLT2 inhibitors group was matched to another T2DM individual that did not apply SGLT2 inhibitors, and the later population constituted the control group. The propensity score-matching (PSM) method was used in this study for matching the two groups by adjusting the demographic, medical and disease factors. After the whole selection process, a total of 41724 T2DM patients were enrolled in the SGLT2 inhibitors group and control group, respectively. The flowchart of selection is declared in Figure 1.

### Major Outcome

The major outcome in this study was newly-developed corneal disease events using the following criteria: (1) the superficial keratopathy and infectious keratitis diagnosis according to ICD-9 diagnostic codes and ICD-10 diagnostic codes, (2) the arrangement of slit-lamp biomicroscopy examination before or at same time of corneal diseases diagnosis by the procedure codes, (3) the application of topical antibiotic eyedrop or ointment after the corneal diseases diagnosis via the ATC codes, (4) the corneal diseases diagnosis was made by an ophthalmologist. Patients in our study were trialed until the fulfilment of these conditions (1) corneal disease diagnosis, (2) individual withdrawn from National Health Insurance program or (3) the terminal of NHIRD which implies the December 31, 2020.

### Demographic and Predisposing Factors

In the current study, we enrolled the effect of specific demographic data, co-morbidities and medications in statistical analysis to adjust the influence of predisposing factors on corneal disease development: age, sex, type of occupation, T2DM duration, hypertension, hyperlipidemia, coronary heart disease (CHD), systemic lupus erythematosus, rheumatoid arthritis, Sjogren syndrome and dry eye disease according to the ICD-9 and ICD-10 diagnostic codes in the NHIRD. In addition, the adapted diabetes complications severity index (aDCSI) which incorporate the presence of diabetic retinopathy, diabetic nephropathy, diabetic neuropathy, ischemic stroke, hemorrhagic stroke, acute myocardial infarction, heart failure, diabetic ketoacidosis, hyperosmolar hyperglycemic syndrome, and end-stage renal disease according to the ICD-9 and ICD-10 diagnostic codes in the NHIRD to reflect the severity of T2DM. Besides, several T2DM-related medications including biguanides, sulfonylureas, alpha glucosidase inhibitors, thiazolidinediones, dipeptidyl peptidase-4 (DPP4) inhibitors, and insulin were also considered in the PSM procedure and statistical analysis. To promise the durations of predisposing factors in this research are long enough to affect the risk of corneal disease occurrence, only the disorders/medical prescriptions which with an interval longer than two years before the index date were included in the statistical analyses.

### Statistical Analysis

The SAS version 9.4 was exploited in our statistical analyses. Descriptive analyses were applied to demonstrate the demography and co-morbidities between the two groups, and the absolute standardized difference (ASD) was engaged to analyze the difference between the two T2DM groups. After that, the Cox proportional hazard regression was operated to offer the adjusted hazard ratios (aHR) with related 95% confidence intervals (CI) of corneal diseases incidence between the two groups, and the effect of demographic characteristics, co-morbidities and medications were adjusted in the Cox proportional hazard regression. We plotted the Kaplan-Meier curve to present the cumulative incidences of corneal diseases between the two groups. In the subgroup analyses, all the T2DM individuals were categorized by age, sex, aDCSI, T2DM duration, the usage of DPP4 inhibitors, and the application of insulin. Then Cox proportional hazard regression was executed again to analyze the incidences and aHR of corneal diseases in different subgroups. Besides, interaction test was made to present the influence of SGLT2 inhibitors on corneal diseases occurrence in different subgroups. The statistical significance was decided as P < 0.05 in this article and a P value less than 0.0001 was depicted as P < 0.0001.

## Results

The basic information of the SGLT2 inhibitors and control groups are demonstrated in Table 1. The distributions of patient numbers in each age interval were similar (all ASD < 0.1). The male percentage were 65.44 and 65.66 in the SGLT2 inhibitors and control groups, respectively (ASD = 0.0045). About the other factors, the distributions of occupation, T2DM duration, diseases, aDCSI, and the anti-diabetic medications illustrated insignificant difference between the two groups due to the PSM process (all ASD < 0.1) (Table 1).

**Figure 2:**
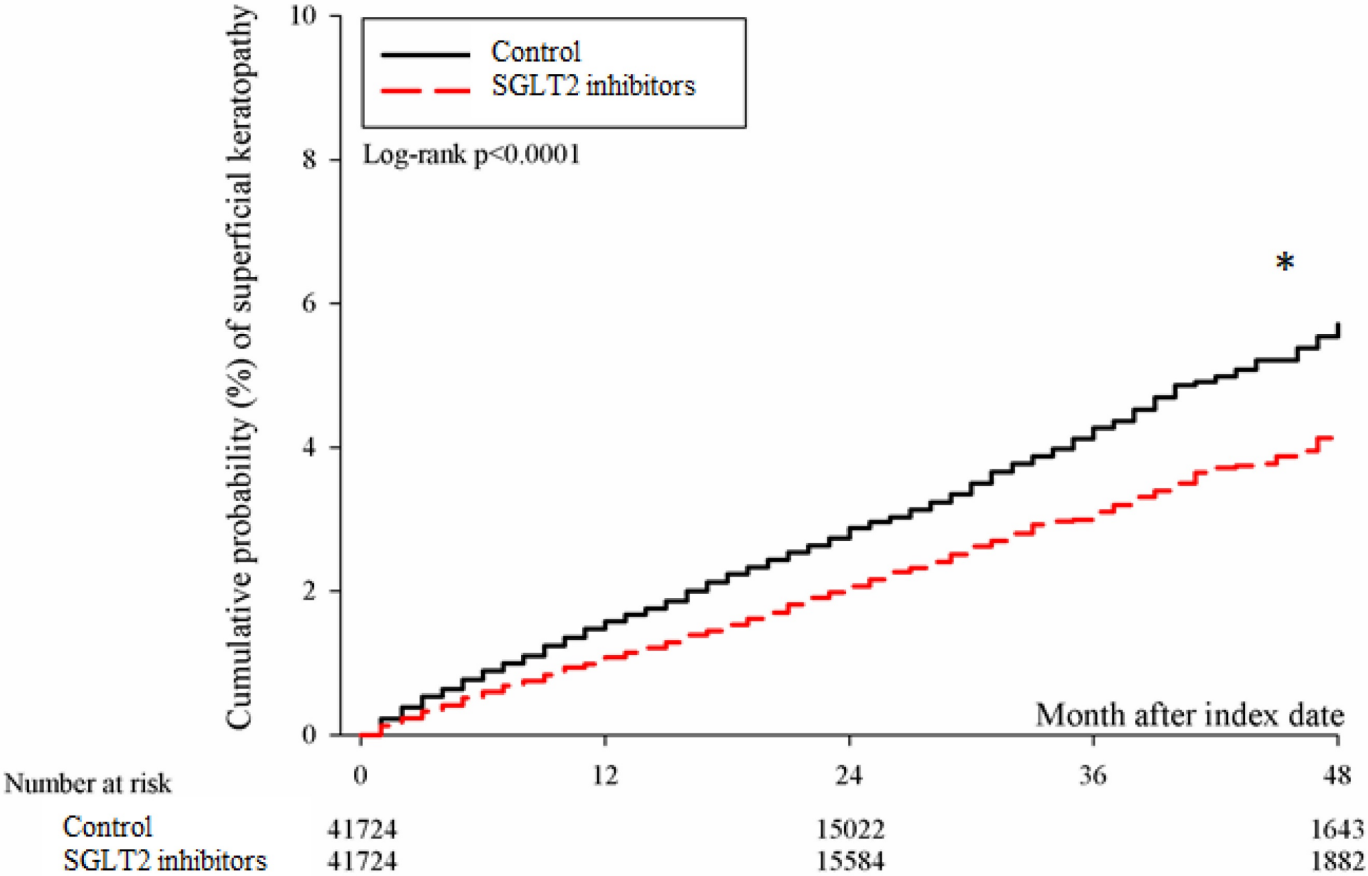
** The cumulative incidence of superficial keratopathy between the two groups.** SGLT2: sodium-glucose cotransporter 2**.** * Denotes significant difference between the two groups.

**Figure 3:**
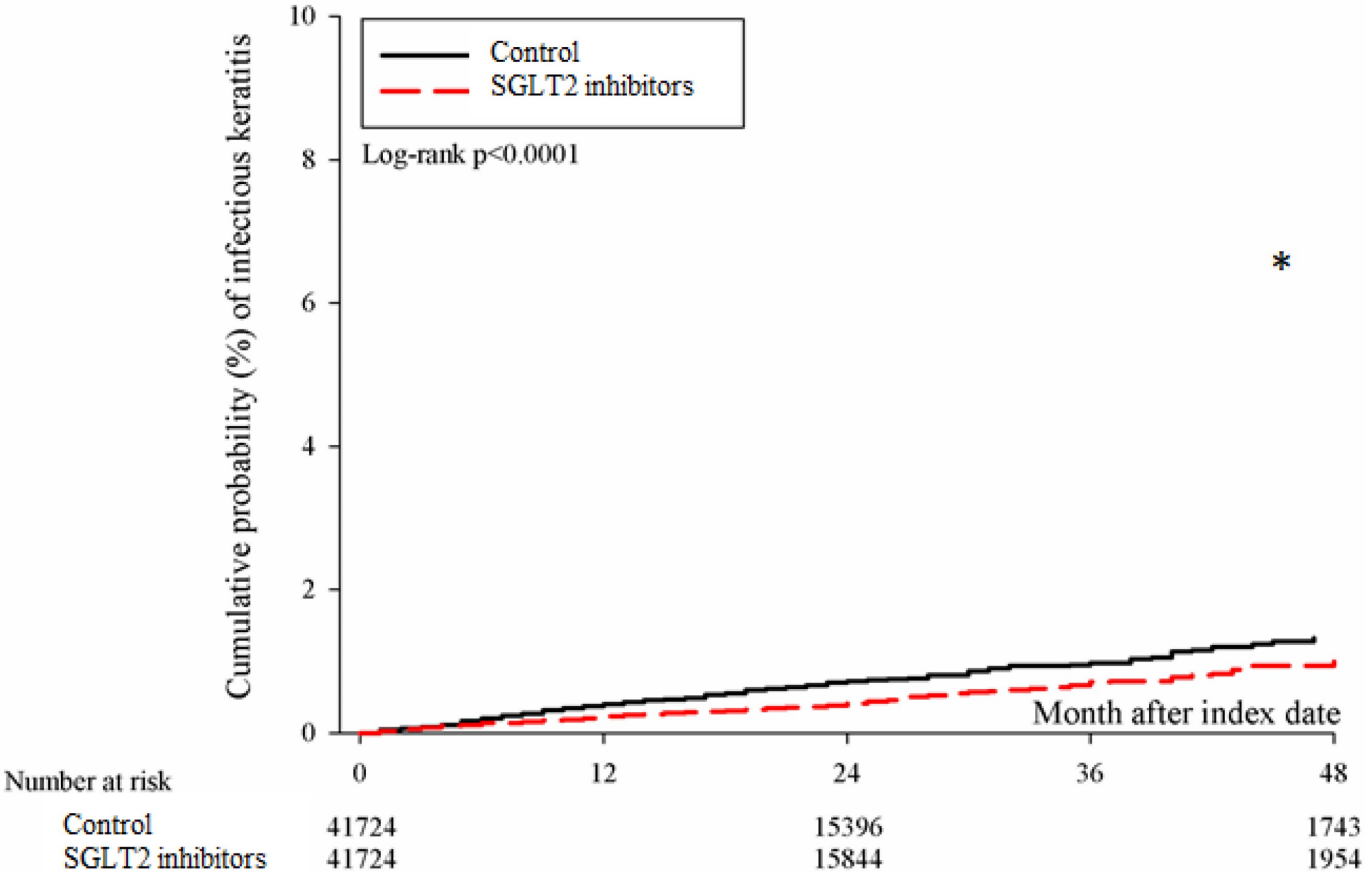
** The cumulative incidence of infectious keratitis between the two groups.** SGLT2: sodium-glucose cotransporter 2**.** * Denotes significant difference between the two groups.

After the follow up period, there were 766 and 1037 episodes of newly-onset superficial keratopathy in the SGLT2 inhibitors and control groups. The SGLT2 inhibitors group showed a significantly lower incidence of superficial keratopathy than the control group (aHR: 0.721, 95% CI: 0.656-0.791, P < 0.0001) after adjusting multiple risk factors (Table 2 and 3). Besides, there were 166 and 251 infectious keratitis events in the SGLT2 inhibitors and control groups, and the patients in SGLT2 inhibitors group also revealed a significantly lower infectious keratitis incidence than the control group according to the Cox proportional hazard regression (aHR: 0.654, 95% CI: 0.537-0.796, P < 0.0001) (Table 2 and 3). About other factors and the development of corneal diseases, the male sex, age between 40 to 70, dry eye disease and aDCSI higher than 1 are correlated to higher incidence of superficial keratopathy development (all P < 0.05) (Table 3). However, there was no other disease or medication factors that influence the incidence of infectious keratitis (all P > 0.05) (Table 4). The cumulative incidences of both superficial keratopathy and infectious keratitis were significantly lower in the SGLT2 inhibitors group than the control group (both P < 0.0001) (Figure 2 and 3).

In the subgroup analyses, the usage of SGLT2 inhibitors correlated to a grossly lower incidences of superficial keratopathy and infectious keratitis compared to the non-SGLT2 inhibitors users (all aHRs < 1), although several SGLT2 inhibitors subgroups showed a similar incidence of infectious keratitis as non-SGLT2 inhibitors population (Table 5). The interaction test demonstrated that the effects of SGLT2 inhibitors on superficial keratopathy development were similar in all subgroups despite the factors of stratification (all P > 0.05), and same results were observed regarding the relationship between SGLT2 inhibitors usage and infectious keratitis development (all P > 0.05) (Table 5).

## Discussion

Briefly, the SGLT2 inhibitors present a significantly protective effect on the development of superficial keratopathy and infectious keratitis after adjusting many potential risk factors. In addition, this effect was universal in patients with different demography, T2DM severity and medication usages and correlated to the duration of SGLT2 inhibitors usage. Besides, age older than 70 years, male sex, higher diabetic severity and the presence of dry eye disease associates with higher chance of superficial keratopathy development.

The SGLT2 inhibitors have demonstrated significant effect on hyperglycemia retardation and protection ability on several organs including malignancy 6, 18, 19. The incidence and severity of CHD can be reduced via the application of SGLT2 inhibitors in which the SGLT2 can decrease the autosis of cardiac cell and the subsequent myocardial infarction episode in the experimental model 7, 20. Besides, the usage of SGLT2 inhibitors is associated with lower rate of major cardiovascular event in patients with atherosclerotic cardiovascular disease 21. In addition, the progression and severity of congestion heart failure can be retarded by the utilization of SGLT2 inhibitors whether the ejection function was preserved or not 9. Except for the cardiovascular system, the urinary creatinine level and the hyperkalemia status in chronic kidney disease was reduced by SGLT2 inhibitors compared to the population without SGLT2 inhibitors usage 4, 9. Besides, the rate of end-stage renal disease was also significantly lower in individuals with chronic kidney disease who took SGLT2 inhibitors 21. About the possible mechanism of SGLT2 inhibitors for its cardiorenal protection, the suppression of inflammation and oxidative stress may be the main reasons 3, 22, 23. The SGLT2 inhibitors can reduce the release of inflammatory cytokine and adipose tissue-mediated inflammation for the heart and kidney 24, 25. In addition, the oxidative stress can be reduced after the application of SGLT2 inhibitors in the myocardial and diabetic kidney disease model 26, 27. Concerning the corneal diseases, the inflammation of meibomian gland is one of the several mechanisms for superficial keratopathy development 28. Moreover, the expression of inflammatory cytokine in ocular surface would elevate the risk of infectious keratitis 29. And the oxidative stress would also increase in the injured ocular surface like the keratitis. Because the SGLT2 inhibitors own anti-inflammatory and antioxidant abilities and demonstrates protective function on several of tissues 24, 30, we speculated that the usage of SGLT2 inhibitors may reduce the ocular inflammation and related corneal diseases. The above concept was partially supported by the findings of our study.

In this study, the prescription of SGLT2 inhibitors on patients with T2DM was correlated to significantly lower incidence of superficial keratopathy and infectious keratitis. To our knowledge, this may be a preliminary experience to depict the correlation between SGLT2 inhibitors usage and incidence of corneal diseases. A previous publication showed insignificant correlation between the SGLT2 inhibitors usage and corneal disease while the few corneal disease events might cause statistical bias 31. In this study, the patient numbers are relative adequate and patients with previous severe corneal diseases such as previous infectious keratitis and corneal opacity due to injury or neovascularization were excluded from this study. Besides, the effect of several possible risk factors for corneal diseases development including the rheumatic arthritis, systemic lupus erythematous, dry eye disease and Sjogren syndrome were adjusted in the Cox proportional hazard regression. On the other hand, the T2DM duration, T2DM-related complications and T2DM-associated medications were all adjusted in the PSM procedure and statistical model. Consequently, the SGLT2 inhibitors could be the independent protective factor for the incidence of superficial keratopathy and infectious keratitis in T2DM patients. In previous study, the inflammatory cytokine interleukin-1 and matrix metalloproteinase-9 were elevated in the patients with keratitis 32, and inflammation response can be suppressed in the diabetic retinopathy by SGLT2 inhibitors 33. Besides, the application of anti-inflammatory agent like the corticosteroid can reduced the severity of infectious keratitis under proper antibiotic coverage 34. On the other side, the SGLT2 inhibitors can suppress the cardiac cell death 3, and the injury of corneal epithelium could lead to infectious keratitis 29. Accordingly, it is reasonable that the application of SGLT2 inhibitors suppress the inflammation, glucose-induced oxidative stress and cell death 3, 23, 35, thus the development of both superficial keratopathy and infectious keratitis become fewer. Furthermore, the lower cumulative probability of both superficial keratopathy and infectious keratitis in the SGLT2 inhibitors group suggested a long-term SGLT2 inhibitors usage concerning the cornea.

In the subgroup analyses, the protective effect of SGLT2 inhibitors on the superficial keratopathy and infectious keratitis developments were marginally stronger in the young T2DM population. There was rare study to present such phenomenon. About the effectiveness of SGLT2 inhibitors on different age interval, the SGLT2 inhibitors produced better therapeutic outcome in T2DM individuals younger than 40-year-old compared to their older counterpart which may result from the better urinary glucose excretion in the young group 36. Besides, the application of SGLT2 inhibitors cause a significant reduction of newly-onset arrhythmias in patients younger than 60 years old while such significance was not observed in those aged over 60 years old 37. Accordingly, the young population might be silently benefited from SGLT inhibitors concerning the superficial keratopathy and infectious keratitis developments. On the other hand, the sex, T2DM-related complications and T2DM medications including DPP4 inhibitors and insulin did not alter the correlation between SGLT2 inhibitors application and the development of corneal diseases. In previous publications, there was no evidence that the different sex or numbers of T2DM complications would influence the control effect of SGLT2 inhibitors on cardiovascular diseases 38, 39. The results of our study corresponded to the previous literatures. Besides, the T2DM duration and T2DM medications that could reflect the glycemic levels such as DPP4 inhibitors and insulin did not alter the effect of SGLT2 inhibitors on corneal diseases development, which further support the universal protective effect of SGLT2 inhibitors on corneal disorders.

Concerning the incidence of T2DM and corneal diseases throughout the world, the T2DM affected more than 8 percent of patients worldwide and the highest incidence was found in the North American region with an incidence of 13 percent 1. And it was estimated that the incidence of T2DM in North American region will reach 14.8 percent in the year of 2045 1. The corneal diseases, although not as prevalent as the T2DM, is a major ophthalmic disorder with an incidence of 2.5 to 799 per 100,000 per population-year in previous literature 29. In the patients diagnosed with corneal diseases, the corneal opacity may develop eventually which is 5th leading cause of blindness and severe visual impairment globally 29. In addition, the T2DM itself is associated with the higher incidence of corneal diseases which may because the hyperglycemic status and related inflammatory response would damage the corneal epithelium and corneal nerve 40. Because both the T2DM and corneal diseases are not uncommon diseases and the presence of T2DM could elevate the risk of corneal diseases which may cause blindness 29, 40, 41, the T2DM therapy that correlates to lower risk of corneal diseases development might be illustrated.

Several limitations presented in the current study. Firstly, the application of claimed data in this study let some important information involving the blood glucose level in T2DM, the glycated hemoglobin level in T2DM, the exact etiology of corneal diseases (i.e. trauma, dry eye related, bacterial infection, fungal infection), the external eye photography of corneal diseases, the fluorescein stain result of corneal disease, the culture result of the corneal disease if existed, the therapeutic outcome of corneal disease and the recurrence of corneal disease is inaccessible. Besides, the retrospective design of our study may decrease the homogeneity of our study population although we used the PSM method to standardize the general condition of the study population. In addition, whether the correlation between SGLT2 inhibitors application and lower incidence of infectious keratitis was resulted from the inflammation suppression or the epithelial barrier preservation cannot be evaluated. Finally, roughly all the participants in the current study were Taiwanese, thus the external validity of this study for other ethnicity is reduced.

In conclusion, the usage of SGLT2 inhibitors in T2DM patients is correlated to a significantly lower incidence of superficial keratopathy and infectious keratitis. Furthermore, the protective phenomenon of SGLT2 inhibitors on corneal disease is positively associated with longer duration of SGLT2 inhibitors application. Consequently, the utilization of SGLT2 inhibitors may be recommended for the T2DM patients with established predisposing factor of corneal disease. Further large-scale prospective study to evaluate the correlation between SGLT2 inhibitors application and the therapeutic outcome of corneal diseases is mandatory.

## Figures and Tables

**Table 1:** The characteristic in SGLT2 inhibitors group and matched diabetes population

Characters	Control group (N=41724)	SGLT-2 inhibitors group (N=41724)	ASD
Age			
20-39	6341 (15.20%)	6193 (14.84%)	0.0099
40-49	11850 (28.40%)	11644 (27.91%)	0.0110
50-59	13410 (32.14%)	13421 (32.17%)	0.0006
60-69	8146 (19.52%)	8313 (19.92%)	0.0101
70-79	1666 (3.99%)	1810 (4.34%)	0.0173
>= 80	311 (0.75%)	343 (0.82%)	0.0087
Sex			
Male	27394 (65.66%)	27305 (65.44%)	0.0045
Female	14330 (34.34%)	14419 (34.56%)	0.0045
Occupation			
Government employee	1569 (3.76%)	1605 (3.85%)	0.0045
Worker	27734 (66.47%)	27416 (65.71%)	0.0161
Farmer and fisherman	4060 (9.73%)	4201 (10.07%)	0.0113
Low income	495 (1.19%)	517 (1.24%)	0.0048
Others	7866 (18.85%)	7985 (19.14%)	0.0073
T2DM duration			
0-6 months	11325 (27.14%)	11261 (26.99%)	0.0035
6-12 months	6115 (14.66%)	4705 (11.28%)	0.1007
12-24 months	8809 (21.11%)	8854 (21.22%)	0.0026
24-36 months	6479 (15.53%)	7129 (17.09%)	0.0422
36-48 months	8996 (21.56%)	9775 (23.43%)	0.0447
Co-morbidity			
Hypertension	21658 (51.91%)	21720 (52.06%)	0.0030
CHD	4370 (10.47%)	4773 (11.44%)	0.0309
Hyperlipidemia	29101 (69.75%)	28708 (68.80%)	0.0204
Rheumatoid arthritis	124 (0.30%)	155 (0.37%)	0.0129
Systemic lupus erythematosus	32 (0.08%)	34 (0.08%)	0.0017
Sjogren syndrome	105 (0.25%)	119 (0.29%)	0.0065
Dry eye disease	14360 (16.58%)	6526 (15.07%)	0.0084
aDCSI score			
0	25250 (60.52%)	24573 (58.89%)	0.0331
1	8953 (21.46%)	9070 (21.74%)	0.0068
2	5515 (13.22%)	5928 (14.21%)	0.0288
≥ 3	2006 (4.81%)	2153 (5.16%)	0.0162
Co-medication			
Biguanides	37381 (89.59%)	37160 (89.06%)	0.0172
Sulfonylureas	17774 (42.60%)	17874 (42.84%)	0.0048
Alpha glucosidase inhibitors	2441 (5.85%)	2604 (6.24%)	0.0164
Thiazolidinediones	3357 (8.05%)	3612 (8.66%)	0.0221
DPP4 inhibitors	16732 (40.10%)	16822 (40.32%)	0.0044
Insulin	3199 (7.67%)	3410 (8.17%)	0.0187

aDCSI: adapted diabetes complications severity index, ASD: absolute standard difference, CHD: coronary heart disease, DPP4: dipeptidyl peptidase-4, N: number, SGLT2: sodium-glucose cotransporter 2, T2DM: type 2 diabetes mellitus.

**Table 2:** The risk of superficial keratopathy between SGLT2 inhibitors and control groups

Study outcome	Control group	SGLT-2 inhibitors group
**Superficial keratopathy**		
Person-months	839013	859035
Event	1037	766
Incidence rate^#^ (95% CI)	12.36 (11.63-13.14)	8.92 (8.31-9.57)
Crude HR (95% CI)	Reference	0.722* (0.658-0.793)
aHR (95% CI)	Reference	0.721* (0.656-0.791)
**Infectious keratitis**		
Person-months	852489	868283
Event	251	166
Incidence rate (95% CI)	2.94 (2.60-3.33)	1.91 (1.64-2.23)
Crude HR (95% CI)	Reference	0.650* (0.534-0.791)
aHR (95% CI)	Reference	0.654* (0.537-0.796)

aHR: adjusted hazard ratio, CI: confidence interval ^#^ Incidence rate: the event case per 10000 person-months * Denotes significant difference between the two groups

**Table 3:** The effect of each parameter on the development of superficial keratopathy

Parameter	aHR (95% CI)	P
SGLT2 (reference: Non-SGLT2)	0.721 (0.656-0.791)	< 0.0001*
Sex (reference: Female)		
Male	0.827 (0.752-0.910)	< 0.0001*
Age (reference: 20-39)		
40-49	0.874 (0.764-1.000)	0.0496*
50-59	0.802 (0.699-0.921)	0.0017*
60-69	0.792 (0.675-0.930)	0.0044*
70-79	0.897 (0.680-1.184)	0.4438
>= 80	0.430 (0.177-1.046)	0.0626
Occupation (reference: Worker)		
Government employee	0.819 (0.626-1.071)	0.1444
Farmer and fisherman	1.184 (0.984-1.425)	0.0728
Low-income	1.065 (0.710-1.598)	0.7611
Others	0.913 (0.800-1.042)	0.1760
T2DM duration (reference: 0-6)		
6-12 months	0.895 (0.772-1.038)	0.1435
12-24 months	0.990 (0.871-1.124)	0.8731
24-36 months	0.915 (0.785-1.066)	0.2548
36-48 months	0.872 (0.727-1.046)	0.1394
Co-morbidity		
Hypertension	0.974 (0.886-1.071)	0.5835
CHD	0.987 (0.835-1.167)	0.8815
Hyperlipidemia	1.055 (0.954-1.168)	0.2977
Ischemic stroke	0.876 (0.657-1.167)	0.3649
Rheumatoid arthritis	0.671 (0.251-1.795)	0.4263
Systemic lupus erythematosus	0.571 (0.080-4.088)	0.5769
Sjogren syndrome	1.332 (0.595-2.983)	0.4857
Dry eye disease	1.233 (1.075-1.963)	0.0035*
aDCSI score (reference: 0)		
1	1.149 (1.017-1.298)	0.0256*
2	1.175 (1.009-1.367)	0.0374*
>= 3	1.091 (1.043-1.411)	0.0492*
Co-medication		
Biguanides	1.013 (0.863-1.188)	0.8784
Sulfonylureas	1.038 (0.941-1.146)	0.4563
Alpha glucosidase inhibitors	0.861 (0.699-1.061)	0.1595
Thiazolidinediones	1.007 (0.837-1.211)	0.9400
DPP4 inhibitors	0.906 (0.817-1.004)	0.0607
Insulin	1.023 (0.863-1.214)	0.7915

aDCSI: adapted diabetes complications severity index, aHR: adjusted hazard ratio, CHD: coronary heart disease, CI: confidence interval, DPP4: dipeptidyl peptidase-4, SGLT2: sodium-glucose cotransporter 2, T2DM: type 2 diabetes mellitus. * Denotes significant association to the development of superficial keratopathy

**Table 4:** The effect of each parameter on the development of infectious keratitis

Parameter	aHR (95% CI)	P
SGLT2 (reference: Non-SGLT2)	0.654 (0.537-0.796)	< 0.0001
Sex (reference: Female)		
Male	1.007 (0.822-1.235)	0.9427
Age (reference: 20-39)		
40-49	0.938 (0.716-1.229)	0.6434
50-59	0.761 (0.572-1.012)	0.0605
60-69	0.704 (0.500-1.090)	0.0633
70-79	0.890 (0.497-1.593)	0.6951
>= 80	0.810 (0.197-3.342)	0.7712
Occupation (reference: Worker)		
Government employee	0.507 (0.251-1.025)	0.0587
Farmer and fisherman	0.804 (0.517-1.249)	0.3314
Low-income	0.394 (0.098-1.587)	0.1902
Others	1.277 (0.998-1.630)	0.0511
DM duration (reference: 0-6)		
6-12 months	1.239 (0.920-1.667)	0.1578
12-24 months	1.185 (0.908-1.547)	0.2109
24-36 months	1.089 (0.791-1.498)	0.6013
36-48 months	0.899 (0.602-1.342)	0.6019
Co-morbidity		
Hypertension	0.991 (0.813-1.207)	0.9257
CHD	0.977 (0.678-1.409)	0.9027
Hyperlipidemia	0.848 (0.692-1.040)	0.1141
Ischemic stroke	0.961 (0.533-1.733)	0.8956
Rheumatoid arthritis	1.530 (0.377-6.203)	0.5515
Systemic lupus erythematosus		
Sjogren syndrome	1.926 (0.475-7.805)	0.3587
Dry eye disease	0.288 (0.040-2.048)	0.2134
aDCSI score (reference: 0)		
1	0.964 (0.743-1.252)	0.7857
2	0.862 (0.615-1.208)	0.3877
>= 3	1.147 (0.694-1.895)	0.5936
Co-medication		
Biguanides	0.913 (0.658-1.268)	0.5873
Sulfonylureas	0.969 (0.789-1.190)	0.7621
Alpha glucosidase inhibitors	1.222 (0.839-1.779)	0.2967
Thiazolidinediones	0.525 (0.317-1.868)	0.3921
DPP4 inhibitors	1.039 (0.841-1.283)	0.7214
Insulin	0.962 (0.673-1.376)	0.8319

aDCSI: adapted diabetes complications severity index, aHR: adjusted hazard ratio, CHD: coronary heart disease, CI: confidence interval, DPP4: dipeptidyl peptidase-4, SGLT2: sodium-glucose cotransporter 2, T2DM: type 2 diabetes mellitus. * Denotes significant association to the development of infectious keratitis

**Table 5:** The subgroup analyses for the risk of corneal diseases development in patients with SGLT2 inhibitors treatment stratified by different parameters

Subgroup	Risk for superficial keratopathy		Risk for Infectious keratitis	
	aHR (95% CI)	P for interaction	aHR (95% CI)	P for interaction
Age		0.0951		0.2710
20-49	0.672 (0.595-0.758)		0.619 (0.483-0.793)	
50-69	0.790 (0.697-0.894)		0.813 (0.617-1.071)	
≥ 70	1.028 (0.672-1.572)		0.988 (0.382-2.552)	
Sex		0.0888		0.7837
Male	0.781 (0.701-0.871)		0.709 (0.568-0.883)	
Female	0.670 (0.584-0.768)		0.708 (0.518-0.968)	
aDCSI		0.7676		0.8386
0	0.730 (0.655-0.814)		0.732 (0.583-0.919)	
1	0.776 (0.646-0.932)		0.742 (0.503-1.095)	
2	0.722 (0.570-0.914)		0.611 (0.356-1.049)	
≥3	0.628 (0.412-0.957)		0.585 (0.241-1.420)	
T2DM duration		0.8199		0.3885
0-6 months	0.670 (0.581-0.772)		0.562 (0.409-0.773)	
6-12 months	0.786 (0.627-0.984)		0.808 (0.519-1.256)	
12-24 months	0.733 (0.617-0.871)		0.674 (0.470-0.966)	
24-36 months	0.775 (0.618-0.973)		0.963 (0.604-1.534)	
36-48 months	0.685 (0.526-0.892)		0.704 (0.383-1.295)	
DPP4 inhibitors		0.9693		0.7514
Without	0.723 (0.653-0.801)		0.692 (0.552-0.867)	
With	0.695 (0.595-0.812)		0.710 (0.523-0.964)	
Insulin		0.4048		0.6867
Without	0.713 (0.652-0.779)		0.700 (0.580-0.844)	
With	0.823 (0.610-1.111)		0.763 (0.395-1.475)	

aDCSI: adapted diabetes complications severity index, aHR: adjusted hazard ratio, CI: confidence interval, DPP4: dipeptidyl peptidase-4, T2DM: type 2 diabetes mellitus.
